# An Ergonomic Comparison of Data Entry Work Using a Keyboard vs. Touch Screen Input Device While Standing and Sitting

**DOI:** 10.4172/2165-7556.s4-007

**Published:** 2014-07-01

**Authors:** KG Davis, MJ Hammer, SE Kotowski, A Bhattacharya

**Affiliations:** 1 Low Back Biomechanics and Workplace Stress Laboratory, Department of Environmental Health, University of Cincinnati, Cincinnati, OH, USA; 2 College of Allied Health Sciences, University of Cincinnati, Cincinnati, OH, USA; 3 Department of Environmental Health, University of Cincinnati, Cincinnati, OH, USA

**Keywords:** Call centers, Musculoskeletal disorders, Joint postures, Discomfort, Usability, Upper extremity

## Abstract

Data entry is a common practice in many facilities throughout the world today. Data entry employees are required to sit at a computer, communicate on the phone, and enter data into the computer through keyboard and mouse clicks for long periods of time. The study objective was to evaluate the postures and discomfort experienced when utilizing a keyboard or a touch screen for processing call center data while in seated and standing positions. Twenty subjects completed simulated food order entry where body postures were measured by a motion capture system and body discomfort and usability were measured by questionnaire. Overall, the results indicated that two conditions resulted in more neutral postures, lower joint velocities, and lowest discomfort in the upper extremities compared to traditional keyboard condition. The best conditions were angled touch screen input device when seated at the lower work surface and standing at the high work surface. These conditions were also the preferred conditions for data entry, as indicated by usability and ordered preference. Overall, touch screens put the worker in working postures that reduced the discomfort response to short term exposure. Further, it is important to accommodate the individual with a proper height of work surface when performing data entry either standing or sitting. With call centers prevalent in many industries around the world, touch screen data entry has the potential to reduce the discomfort workers experience when performing long periods of static and relentless work activities.

## Introduction

Data entry is a common practice in calls centers in the United States. Call centers have increased in popularity as the service sector has increased its prominence in the world economy. In fact, according to the North American Call Center Report of 2004 by *McDaniel Executive Recruiters* [1], there are currently 50,600 call centers in the U.S. Call centers are also prevalent around the world with more than a million employees worldwide [2–5]. The nature of the work in these call centers is repeated short periods of data entry while individual listening to information provided over the phone. While the periods of entry are short, they routinely occur throughout the work shift, and thus result in long sustained periods of work. Traditionally, call centers are locations where calls are either placed or received in high volume with the intent of sales, marketing, telemarketing, customer service, or technical support [6].

Upper extremity (wrist and hand), neck and back musculoskeletal disorders (MSDs) are very prevalent in call centers [5]. Researchers have reported prevalence of MSDs and symptoms above 40% in these facilities [2,4,5,7]. Several risk factors for MSDs of the upper back and upper extremities are associated with the tasks performed by the call center operators, including high workload (intense levels of call processing) [2,4,7], poorly adjusted work surface heights [4], and workstation layout parameters such as monitor, keyboard, and mouse location [6]. The intense workload commonly faced by the call center operators may be detrimental due to the nature of the work—long periods of entering data with limited breaks [8].

One optional input device that may be conducive to the call center work demands is the touch screen. There are several advantages of touch screens over traditional keyboard entry including: 1) touching a visual display requires less thinking and is a form of direct manipulation that is easy to learn, 2) it is the fastest input device, 3) hand-eye coordination is easier than mice or keyboards, 4) no extra workspace is required, 5) it is durable in public-access and in high-volume usage, and 6) it has ability to be custom designed (9). However, one study reported the use of touch screen input device resulted in increased discomfort in the shoulders, neck, and fingers [9,10].

While it appears that the best angle of use for the touch screen would be dependent upon the anthropometric dimensions of the individual performing the data entry, past studies have indicated that there is no anthropometric-based optimal viewing angle for touch screens. Schultz and associates [11] recommended a range of 30° to 55° from the horizontal. This range was based on 92% of the subjects adjusted the display to an angle within this range. It is also interesting to note that nearly half (46%) adjusted the angle between 44° and 49°. However, the research relied on the subjective perceptions of the individuals rather than quantitative measurement.

To offset the negative effects of data entry on the musculoskeletal system, another alternative may be to introduce variable postures throughout the day by altering between standing and sitting positions. To be effective, the work stations need to be properly adjusted to the individual worker when sitting or standing, as well as accounting for different input devices. Sit-stand workstations have been found to increase the discomfort in the lower legs and hand/wrist [12]. A review by Karakolis and Callaghan [13] found that six out of seven studies found sit-stand workstations decreased in body discomfort, and thus concluded that these workstations were effective in reducing musculoskeletal burden without impacting productivity.

In order to investigate the synergistic effect of alternative input devices (e.g. touch screens) and sit-stand workstations, a laboratory study was conducted to investigate the postural responses and body discomforts. The objective of the study was to determine whole body postures adopted and body discomfort when entering data through two modes—touch screen and keyboard. More specifically, the study compared: 1) keyboard entry to touch screen entry in sitting and standing configurations and 2) touch screen entry under three conditions (vertical, angles at 45° and flat) in sitting and standing configurations. The evaluation of the postural responses with the touch screen and keyboard input modes included evaluation of low and high height work surfaces. The computer mouse is recognized as a viable input device but was not in the scope of our current study.

## Methods

### Study overview

This experimental study evaluated the differences in joint posture and velocity and body discomfort when utilizing a keyboard and touch screen to enter order during a laboratory simulation of food order entry. The impact of touch screen position was evaluated by comparing three different positions: 1) vertical, 2) horizontal, and 3) 45° from horizontal angled position. In addition, the impact of standing was evaluated under two conditions: 1) standing with desk height at traditional height—74 cm and 2) standing height with elevated desk height (dependent on subjects’ self-selection but could be adjusted from 69 cm to 109 cm). A sit-stand table with a pneumatic lift function was used to adjust the desk surface up and down, depending on the anthropometric data and desired position of the subject.

### Participants

Twenty subjects (10 males and 10 females) participated in the study. In order to be eligible to participate, subjects had to fall in the age range of 18–65 years old. All subjects were required to sign a consent form approved by the University of Cincinnati Institutional Review Board (Protocol # 06-09-27-01E). All the subjects were either students, staff members employed at the university, or colleagues of other subjects with limited data entry experience. All subjects had no current musculoskeletal symptoms or previous injuries or surgeries. Eight of the 10 males and 9 of the 10 females were right handed. Table 1 provides a summary of the anthropometric and demographic data.

### Study design

The study was designed to evaluate twelve specific combinations of the keyboard or touch screen input device and the height of the work surface conditions. There were three independent variables: 1) input device (keyboard vs. touch screen), 2) position of touch screen (vertical, horizontal, and angled) which was nested with touch screen input device condition, and 3) working height (sitting with desk at sitting height, standing with desk at sitting height, and standing with desk at standing height). The sitting height desk height was 74 cm, which is the standard height found in the workplace. Work surface height for the standing conditions were selected by the subject and kept consistent for all standing conditions for a given subject.

**Figure 1:**
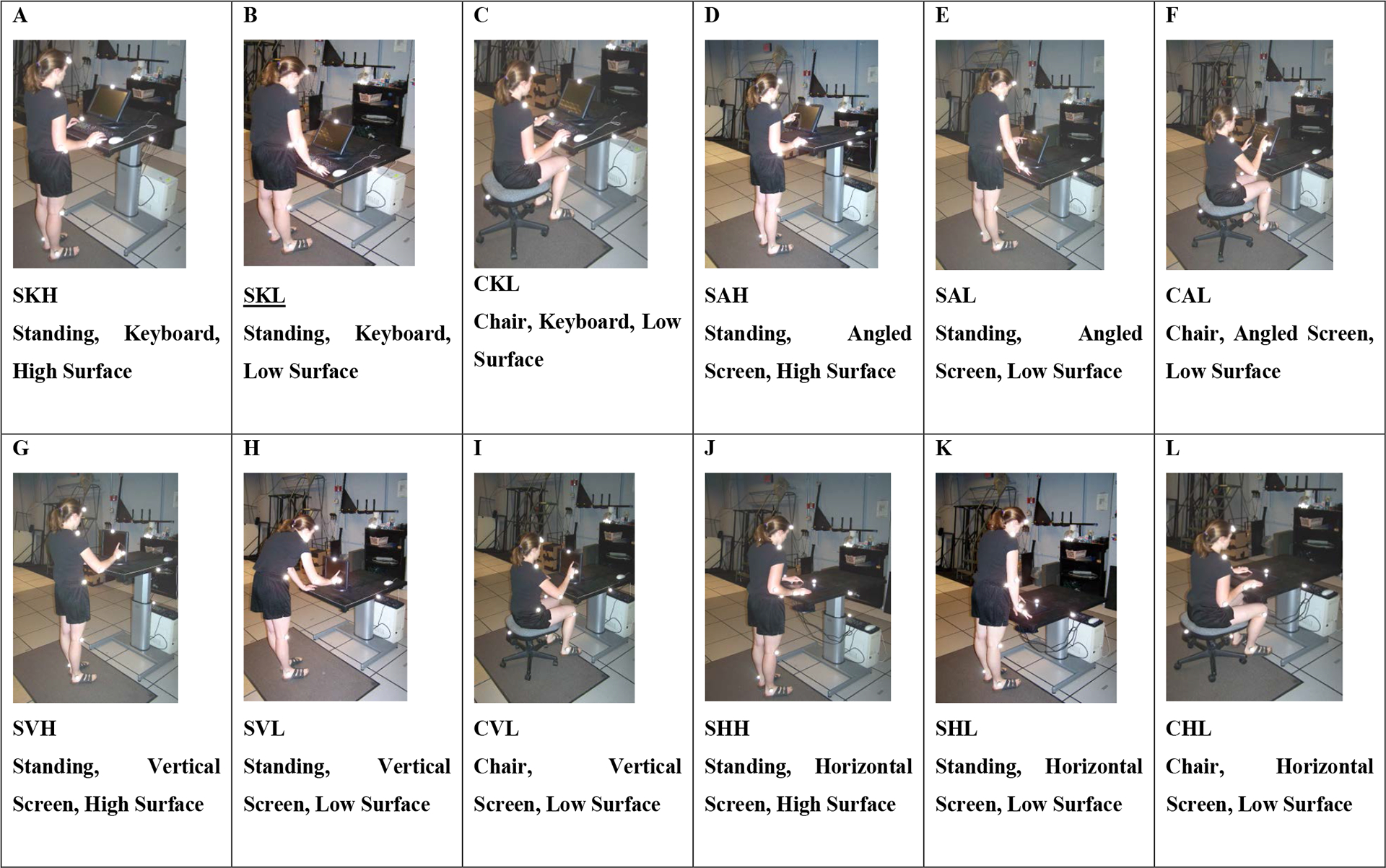
Subject Completing Data Entry in Each of the Twelve Test Conditions.

A counter-balanced and randomized strategy was utilized to select the condition order for each subject to protect from order bias. The three sit-stand conditions (sitting, standing with desk at sitting height, and standing with desk at standing height) were counter-balanced with the input device conditions (keyboard, vertical touch screen, horizontal touch screen, and angled touch screen) being randomly completed within each sit-stand condition. Counter-balancing within the height of work surface and seated/standing conditions reduced the number of times the surface height had to be changed and keep the heights standard for a subject. A photo of each condition is shown in Figure 1.

There were four specific dependent variables evaluated within this study: 1) joint postures, 2) joint velocities; 3) body discomfort; and 4) usability data. Joint postures and velocities were quantified by the Peak Motus Motion Capture System by placing markers on bony landmarks of the body. The markers are digitized and joint postures were identified with velocities being computed by the derivatives of the angles. Body region discomfort was measured by visual analog scale that corresponded to perceived discomfort in the specific body regions immediately following the data entry tasks. The perceived usability of each condition was captured utilizing a standard questionnaire.

### Procedure

After being enrolled into the study, each subject read and signed a consent form. The subjects started with a short practice session (10 minutes in duration) using both the keyboard and touch screen input devices. Under each condition (both practice and actual), a list of orders was read, simulating the processing of a phone order that required the subject to enter the information into the computer via keyboard or touch screen. There was a predetermined script that was read out loud by a researcher (who was sitting out of sight at the other end of the room). For this script, there were 18 different orders (6 easy, 6 moderate, 6 complex) created using various combinations of up to 41 different menu items. The complexity of the orders was determined based on the number of items to be entered (easy = 3 to 5 items, moderate = 6 to 10 items, and complex = 11 to 15 items). Menu items were 18 different drinks, 4 different appetizers, 16 different main courses, and 3 different desserts. The subject was required to enter six orders (two at each complexity level) for each of the twelve conditions, for a total data entry of 72 orders. Each order (or trial) lasted between 20 and 80 seconds depending upon the complexity and performance of the subject. Each condition had the same six orders presented but in a random order to ensure no memorizing of the orders. Orders were either entered using hot function keys on the keyboard or buttons on the touch screen. Each order entry was check upon completion to determine if it was correct by verifying the total cost. If there was an error in entry, the order entry was redone.

### Test Set-up

The complete test set-up included 6 motion-capturing cameras, adjustable sit/stand table, chair (with no back or arms), keyboard, and VDT monitor (38 cm ELO touchscreen, Milpitas, CA) which was used in all conditions. The cameras were part of the Peak Motus Motion Analysis system, where reflective markers were located on bony landmarks, allowing the identification of joint angles and velocities. Each subject had nineteen reflective markers placed on the anatomical joint locations (left and right sides) at the base of the fifth metacarpal, the wrist, elbow, shoulder, hip, knee, ankle, and temple, along with the base of the neck. One additional marker was placed on the subjects’ left thigh to aid in the identification body direction during the digitizing process.

### Joint posture and velocity quantification

The whole body postures and velocities were captured by the Peak Motus Motion Capture System. The system utilizes video capturing capabilities through the use of reflective markers located at bony landmarks that designate specific joints of interest. The joint angles are then calculated by the Motus software. The Peak Motus System has standard data reduction algorithms for editing, filtering, and calculating linear and angular displacements, velocities, and accelerations, which was utilized to calculate joint angles and velocities. The significant joint angles that were of interest included the angle of the upper and lower arms, the wrists, trunk, and neck.

### Quantification of body discomfort

A Body Discomfort Survey was utilized to collect current discomfort in the different body regions. The Body Discomfort Survey assessed the current discomfort levels in nine different body regions including the neck, shoulder, elbow, hand/wrist, upper back, low back, hip, knee, and lower leg/foot. Immediately after completing a specific condition, subjects circled the appropriate pain level on a 0 to 10 visual analog scale, with 0 being no pain, and 10 being most severe pain imaginable. The visual analog scale has been shown to be valid and reliable to determine current pain levels [14,15].

### Quantification of usability

A Usability Survey, which was adapted from the IBM Usability Survey [16], asked eighteen questions about the usability of the current workstation condition. Usability refers to the ease of use and learnability of the workstation. The content of the questions refers to the workstation, data entry device, screen layout, and overall ease of use. Responses for this survey range from 1–7, with 1 being “strongly disagree” and 7 being “strongly agree.” A Usability Index was calculated by averaging all 18 responses.

### Statistical analyses

Descriptive statistics were computed providing means (standard deviations) of the peak posture and motion variables were computed as a function of input device-workstation condition. A repeated measures split-plot analysis of variance (ANOVA) was performed on all continuous dependent variables (joint angles and velocities). For all significant independent variables, post-hoc analyses (Tukey multiple pairwise comparisons) were performed to determine the source of the significant effect(s) (p<0.05). The discomfort and usability variables were analyzed using a non-parametric analysis, Kruskal-Wallis Rank Sum. Statistical Analysis Software—SAS (version 9.1, Carey, NC) was used for all statistical analyses.

## Results

### Joint angles

**Figure 2:**
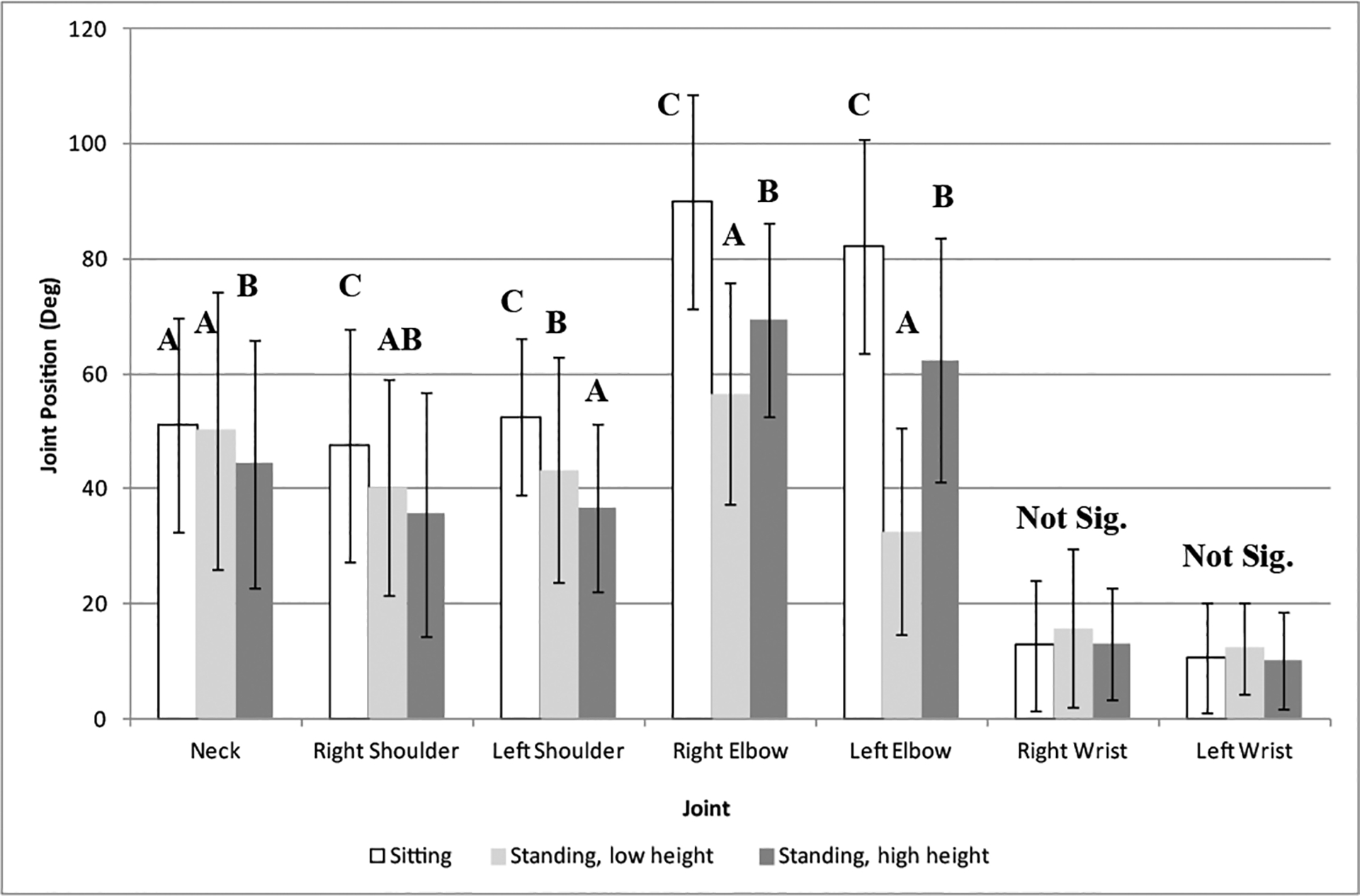
Maximum angles of the right and left elbow, right and left shoulder, right and left wrist, and neck as a function of work surface height (main effect). Different alpha characters indicate significant difference.

It was not surprising that the knee and hip angles were different as a function of work surface height where the standing conditions were closer to 180° as compared to the sitting condition (closer to 90°). There were some small differences in the knee and hip angles for the standing at low surface (mean right and left hip (SD): 160.3° (20.2°) and 158.6° (22.0°); mean right and left knee: 171.5° (9.5°) and 170.3° (8.3°)) and standing at high surface (mean right and left hip (SD): 172.7° (7.5°) and 171.0° (6.9°); mean right and left knee: 172.4° (9.6°) and 170.7° (8.5°)), about 13° for hip and 2° for knee. Other differences were found for the elbows, shoulders, and neck for the different work surface heights (Figure 2). The sitting condition had the most flexed elbow positions (mean right elbow (SD): 97.8° (18.6°); mean left elbow (SD): 90.0° (18.5°)) with the standing at the low height having the most extended elbow position (mean right elbow (SD): 147.4° (18.0°); mean left elbow (SD): 123.5° (19.3°)). Both the sitting and standing at the low height table conditions had greater shoulder flexion than the standing at the higher height table (about 10°). A similar trend was found between the low height work surface (sitting or standing) and standing at the high height for neck (mean low height-sitting (SD): 51.1° (18.6°), mean low height-standing (SD): 50.2° (24.1°), mean high height standing (SD): 44.3° (21.7°)).

**Figure 3:**
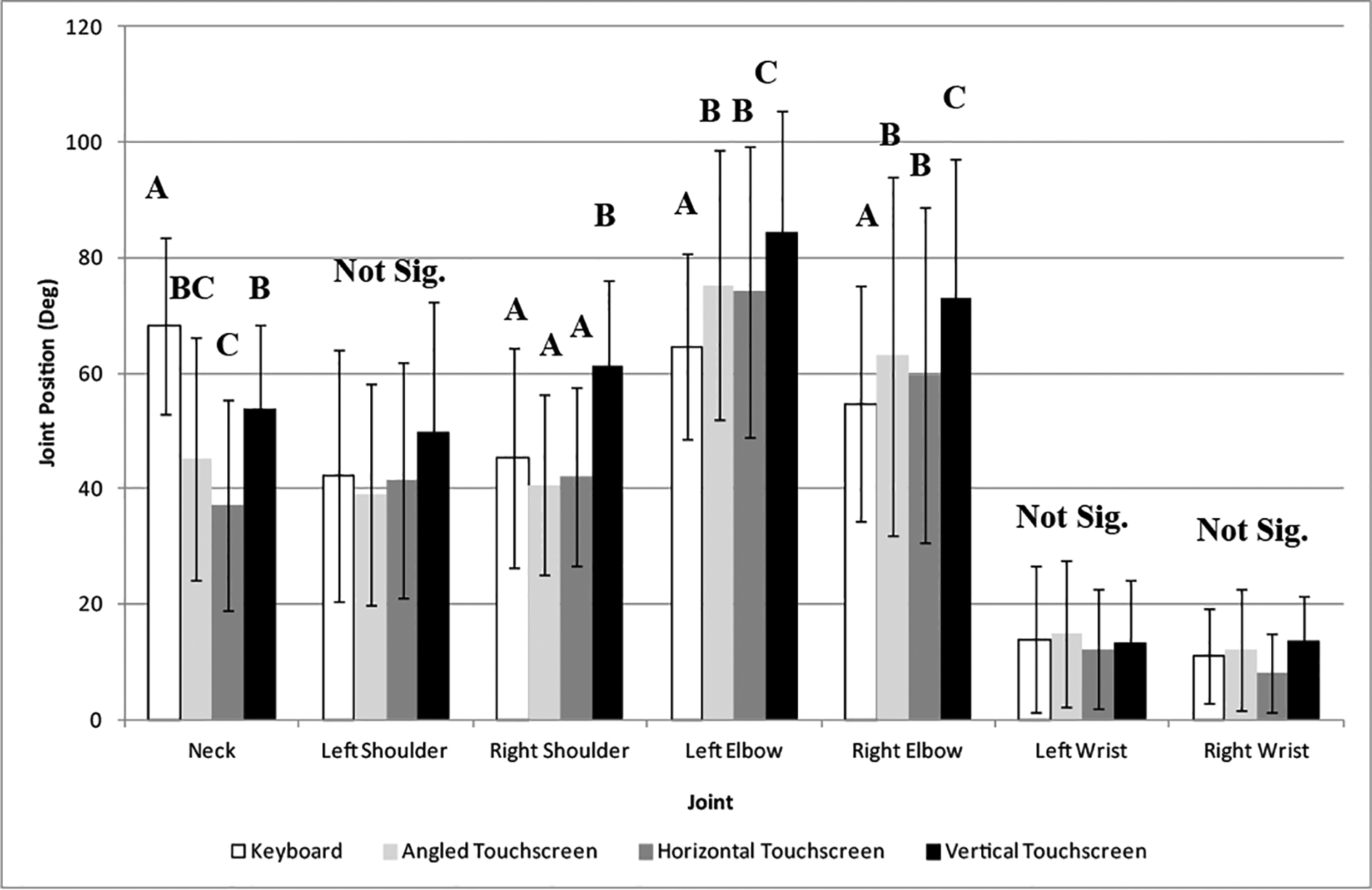
Maximum angles of the right and left elbow, right and left shoulder, right and left wrist, and neck as a function of type of data entry device (main effect). Different alpha characters indicate significant difference.

In Figure 3, the vertical position of the touch screen input device had the most flexed right and left elbows and right shoulder (mean (SD): 72.9° (24.3°), 84.4° (21.1°), and 61.2° (15.0°), respectively) with the other input devices having approximately 60°, 74°, and 42° for right and left elbows and right shoulder, respectively. The highest neck flexion was found for the keyboard conditions (mean (SD): 68.3° (15.2°)) while the least neck flexion was found for horizontal touch screen (mean (SD): 37.1° (18.2°)) (Figure 3).

There were a couple of significant interactions between work surface height and type of input device. The horizontal touch screen and keyboard input devices were found to have the most right wrist flexion when the work surface was low and when standing. Standing at the high surface utilizing these two types of input devices produced the least amount of right wrist flexion (about 5° less). There was little difference between the different work surface heights when using the vertical or angled touch screens (less than 3°). The keyboard and vertical touch screen had significantly more neck flexion, particularly when standing at the low work surface height. In all, the neck flexion was impacted by both the type of input device and the height of the work surface.

Although there was a small difference between males and females for left knee flexion, the difference mainly occurred in the sitting position with the low work surface. As expected from the main effect of surface height for left knee angle, the standing condition had straighter legs. A similar trend was found for left and right hip angle where any difference between males and females occurred for the sitting condition, although this was minimal.

A more complex interaction between gender and work surface height was found for right and left elbow angle. There was a general trend of more extended elbow angles for the standing positions as compared to the sitting condition. Males used a straighter arm (larger elbow angle) posture as compared to females when in the sitting at the low work surface and standing at the high surface. Under the standing high surface, females utilized the extended elbow angle. Under the standing low surface height, the females actually had elbow angles that were straighter (opposite of the other surface height conditions).

### Angular velocities

The lower extremity angular velocities were impacted by the surface height conditions. In general, the sitting at low surface height conditions had lower angular velocities for knee and hip motion. No differences were found between the two standing height conditions. Standing at the high work surface was found to have the lowest elbow angular velocity (about 5 to 8°/s slower). No differences were found between sitting and standing at the low work surface height.

**Figure 4:**
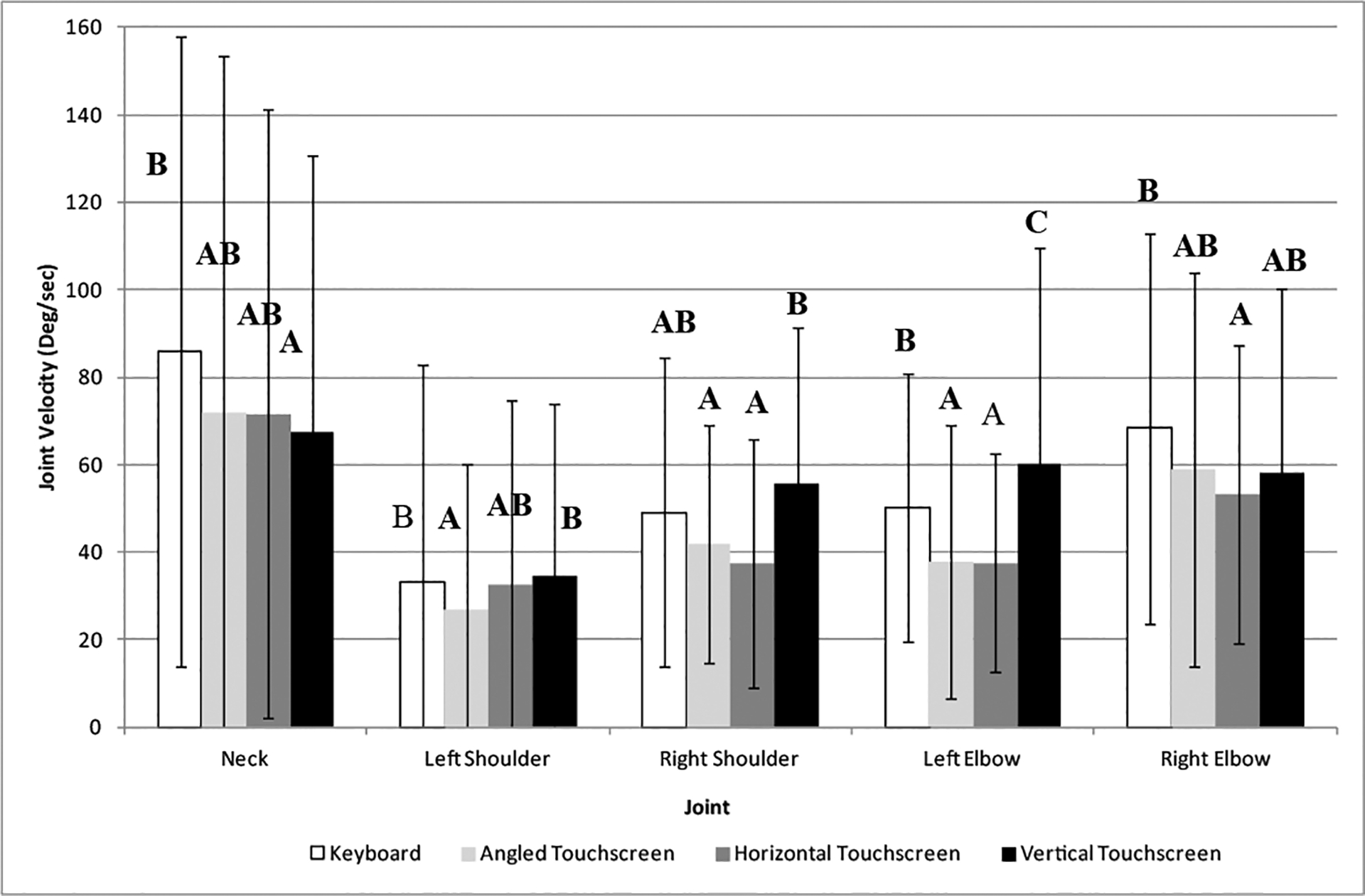
Maximum angular velocities of the right and left upper extremities (elbow and shoulder) and neck as a function of type of data entry device. Different alpha characters indicate significant difference.

In Figure 4, the type of input device impacted the angular velocities of several joints: right knee, right hip, right and left elbow, right and left shoulder, and neck. In general, the keyboard and vertical touch screen had the greatest velocities for all these joints while the angled and horizontal touch screen had the lowest velocities (around 10 to 25°/s between the highest and lowest). The differences between the input devices were influenced by gender. Differences between the input devices were more pronounced for females where the keyboard and vertical touch screen devices produced significantly more velocity in the right knee. A similar effect was found for the left knee where the main difference for the vertical touch screen was due to the females who had more angular velocity than the males (almost 25°/s). Another difference between males and females was noted for the horizontal touch screen condition where females had more angular velocity on the left knee (about 12°/s). Neck velocities were also found to be different between the males and females where females had the higher velocities when using the horizontal touch screen and keyboard input devices but lower velocities when using the angled touch screen. Basically, the interaction indicated that males and females moved their necks differently while utilizing the different input devices.

### Body discomfort

**Figure 5:**
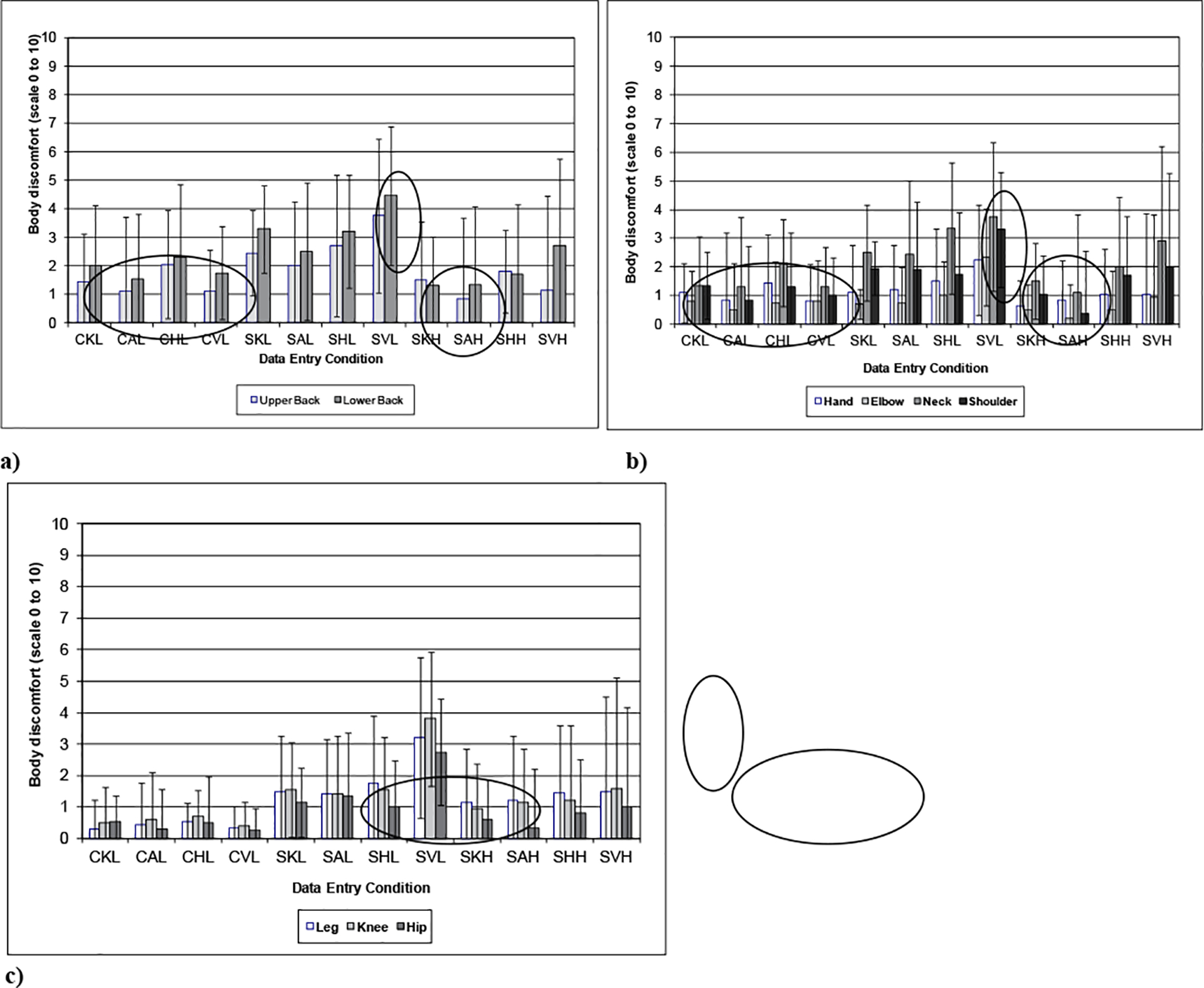
Body discomfort rating for a) upper and lower back, b) upper extremity: hand, elbow, neck, and shoulder and c) lower extremity: legs, knees, and hips as a function of work surface height and type of input device (0= No discomfort 10= Severe discomfort) with conditions: CKL= Chair (Sitting), Keyboard—Low Surface; CAL= Chair (Sitting), Angled Screen—Low surface; CHL= Chair (Sitting), Horizontal Screen—Low surface; CVL= Chair (Sitting), Vertical Screen—Low Surface; SKL= Standing, Keyboard—Low Surface; SAL= Standing, Angled Screen—Low surface; SHL= Standing, Horizontal Screen—Low surface; SKH= Standing, Keyboard—High Surface; SAH= Standing, Angled Screen—High surface; SHH= Standing, Horizontal Screen—High surface; SVH= Standing, Vertical Screen—High Surface.

Figure 5 shows the discomfort in the a) upper and lower back and b) hand, elbow, neck, and shoulder for the different workstation height and type of input device. Standing at the low work surface while entering data with the vertical touch screen resulted in the greatest body discomfort in the lower and upper back. Several other conditions were found to have relatively low upper and lower back discomfort including most of the sitting conditions and the standing at the high work surface height (with the exception of the vertical touch screen). Similar discomfort responses were seen for the hand, elbow, neck, and shoulder. For most of these body regions, the standing at low work height and using the vertical touch screen produced the greatest discomfort. The angled touch screen produced the lowest discomfort, slightly below the horizontal touch screen and keyboard conditions when sitting or standing at the high work surface height. The horizontal touch screen also increased the discomfort when in a sitting posture or standing at the low work surface height.

The discomfort in the legs, knee, and hips also showed a similar trend with the vertical touch screen when standing at the low surface height having the greatest discomfort in these regions. The standing at the low work surface height had slightly more discomfort than the sitting conditions with the two standing heights being approximately the same in discomfort (with the expectation of the vertical touch screen while standing at low work surface height). In all, the vertical touch screen position produced the most discomfort in all body regions while many of the sitting conditions had the least discomfort.

### Usability index

**Figure 6:**
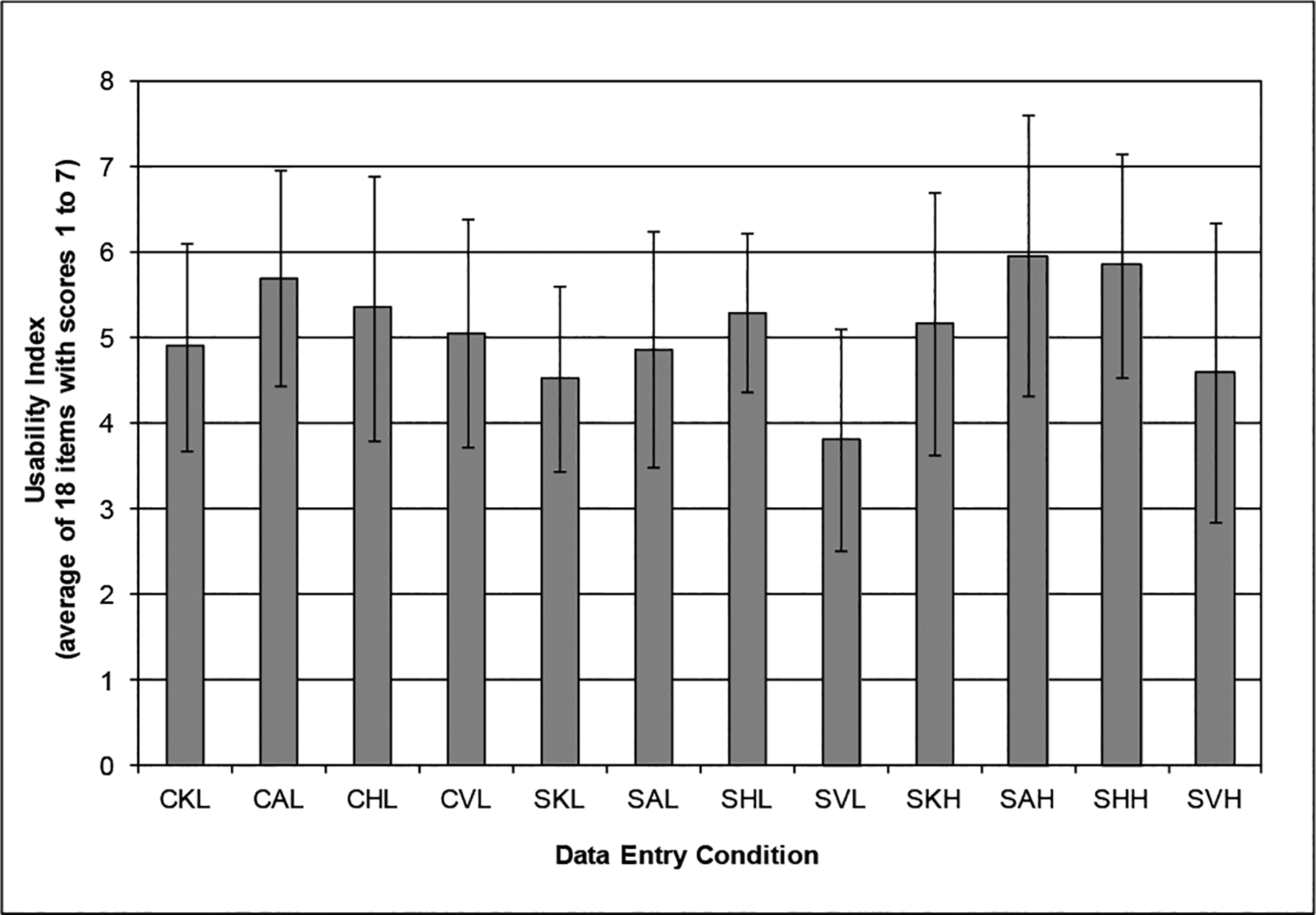
Usability Index as a function of work surface height and type of input device (values between 1 and 7). CKL= Chair (Sitting), Keyboard—Low Surface; CAL= Chair (Sitting) , Angled Screen—Low surface; CHL= Chair (Sitting), Horizontal Screen—Low surface; CVL= Chair (Sitting), Vertical Screen—Low Surface; SKL= Standing, Keyboard—Low Surface; SAL= Standing, Angled Screen—Low surface; SHL= Standing, Horizontal Screen—Low surface; SKH= Standing, Keyboard—High Surface; SAH= Standing, Angled Screen—High surface; SHH= Standing, Horizontal Screen—High surface; SVH= Standing, Vertical Screen—High Surface.

The usability index was greatest for the angled and horizontal touch screens while standing at the high work surface and lowest for the vertical touch screen while standing at the low work surface height (Figure 6). While there were other trends among the usability for the other conditions, none were statistically significantly different from each other. It is worth noting that the vertical touch screen was generally rated as having the lowest usability in all the surface height conditions.

## Discussion

Through evaluation of the postural load, device usability, and current body discomfort, the results indicate that the type of data entry device, position of the data entry device and relative position of the worker (e.g. standing vs. sitting) played a role in the best position when entering data. Table 2 provides a visual summary of the significant results for all the outcome variables. This table uses CKL (sitting with a keyboard and a low surface) as the “reference condition” because this position is the most commonly used. The “up” arrows indicate a significant increase while the “down” arrows indicate a significant decrease. The other boxes with a line through them indicate “no significant difference,” meaning the values did not significantly differ from the reference condition.

In general, the sitting conditions resulted in lower velocities and less awkward postures. The CAL condition (sitting with an angled screen and a low surface) shows good results, with less awkward postures in the shoulder and hips, less joint movement for majority of the body parts, lower body discomfort values, and a higher usability score as compared to the traditional sitting keyboard condition. The CAL condition can be considered one of the “best” options that were tested. Similar results were found for the SAH (standing with angled screen and a high surface). Other conditions that were slightly better than the keyboard data entry option included sitting with the horizontal touch screen on the low surface and to a lesser extent, the sitting with the vertical touch screen on the low surface. The worst condition was standing with the vertical touch screen at the low height where almost all outcome measures were worse than the standard keyboard position. In general, standing at the low work surface was less than optimal, independent of the input device.

Another key issue for call center workers is the prolonged sitting and awkward postures, oftentimes without breaks. Multiple studies have evaluated the effects of exercise breaks and work-rest regimens to reduce the effects of prolonged postures during data entry tasks. Balci, [17] reported “micro” breaks after short periods of work represented a good option to control adverse effects of prolonged computer work. A study by Henning and associates [18] found that the average length of breaks for data entry operators was 27.4 seconds (based on the perception of when they felt they were ready to continue data entry after the break). However, there is no absolute guideline regarding the break time required for adequate rest or recovery to take place [19].

One option of providing breaks may be implementing sit-stand workstations, which could adjust between lower (sitting) and higher (standing) work surfaces. Based on the results, it was clear that workers who stand periodically during the day need a raised work surface to ensure minimal ergonomic stress. The combination of proper work height (adjustable) and angled touch screen input device resulted in the best outcomes. Furthermore, when the participants were asked to rank all 12 conditions, using the angled touch screen while sitting at low surface height and standing at high work surface were ranked as the “most favorite”, indicating a preference for these two options. Anytime one attempts to implement an intervention, it is important to gather buy-in from the worker and these preferences indicate a high level of buy-in for these two work station options. The preference for angled touch screen would support the results of Schultz and associates [11] who found an angled touch screen between 30° and 55° to be perceived as optimal. The position of the current touch screen is in the middle of this range (45°). The current results indicate some biomechanical evidence may be behind the preference of an angled touch screen.

In order to completely understand the results of the current study, there are several limitations that need to be considered. First, all the participants were inexperienced in data entry at a call center. While some subjects did mention that they had previous experience doing similar tasks such as data entry, their experience was limited. Most of the subjects were college students or individuals with a working knowledge of computers and touch screens. Second, there was potential that not everyone was comfortable with using the touch screen or the keyboard due to not being familiar with the system being used. This could have a minor impact on the performance with the different devices but practice should have minimized the effects. Third, many of the independent variables only had limited levels, which may have influenced the biomechanical responses, discomfort ratings, and practical usage of the devices. One example would be the angled touch screen at one position. The angle was based on previously published literature that recommended preferred range of angles [11]. A moderate change in the angle may improve or worsen the responses and should be investigated in the future.

Fourth, the biomechanical evaluation was limited to kinematics of the different joints. More informative results may be obtained by utilization of a biomechanical model that would be able to predict complex loads on all of the joints in the body. However, the current state of biomechanical models makes it difficult to predict accurate loads under the current conditions (e.g. seated postures) and across multiple joints (e.g. only simple models are available across all joints).

Fifth, the findings that tended to favor touch screen data entry may be a reflection of the type of data entry. The items being entered reflected a menu style where either hot keys or buttons on the screen are utilized. While the actual task may be most appropriate to menu style entry, similar results would be expected if the entry was different as the position of the devices would be the same. Further, menu style entry may be more applicable in food industry than traditional data entry. Some circumstances such as entering extensive text may impact the effectiveness of the touch screen. Thus, the results may have limited applicability to the larger industry of call centers that have lots of text entry.

Sixth, the size of the participant (individual anthropometry) could influence the biomechanical responses and corresponding discomforts. While the height of the work surface was a major factor in determining overall discomfort levels, postural load, and usability of the workstation, the subject stature and other height related measurements (e.g. upper torso length) would directly impact the influence of height of the work surface. A taller worker is required to bend farther over or lean forward more, which potentially translates into more biomechanical stress and discomfort. Worker height would have the largest impact when standing and working at the low work surface height. As a result, the best work surface is one that is adjustable.

Another worker related factor that was not taken into account is the body mass index (BMI). With obesity trends on the rise in United States and worldwide [20,21], excessive weight may influence how people perform their jobs in the future and may influence postures. In the current study, there were only a few subjects that would be considered obese with the majority of the individuals being of average height and weight. Therefore, it was hard to draw any conclusions regarding obesity trends in this study. With that being said, obesity could in fact play a role in the postures workers adopt as well as the perceived discomfort when performing the data entry tasks.

## Conclusion

The current study investigated keyboard and touch screen data entry, evaluating the potential trade-offs between kinematic responses, discomfort, and usability. Based on the results in their entirety (e.g. kinematics, discomfort, usability, and error rates), the angled touch screen was found to be equal or better than the traditional keyboard data entry device. It is also important to use a work surface that is at the appropriate height—low height when sitting and high height when standing. The best conditions (out of those investigated) were the angled touch screen when sitting at low height and angled touch screen when standing at high height. As a result, sit-stand tables may be beneficial to call center employees who alter between standing and sitting.

## Figures and Tables

**Table 1:** Demographic and Anthropometric Data for the Participating Subjects (10 males and 10 females).

		Mean	Std dev
Age (years)	Male	25.1	4.1
	Female	31.2	12.5
Height (cm)	Male	177.7	5.5
	Female	164.9	7.9
Weight (kg)	Male	86.5	14.8
	Female	70.3	16.1
Shoulder Height (cm)	Male	148.5	5.9
	Female	137.1	7.1
Elbow Height (cm)	Male	109.5	4.4
	Female	101.4	6.0
Upper Arm Length (cm)	Male	35.6	1.6
	Female	33.8	1.5
Lower Arm Length (cm)	Male	46.7	1.8
	Female	43.3	1.4

**Table 2:** Summary of the results by comparing the reference condition (CKL= Chair (Sitting), Keyboard—Low Surface) to each of the other conditions: CAL= Chair (Sitting), Angled Screen—Low surface; CHL= Chair (Sitting), Horizontal Screen—Low surface; CVL= Chair (Sitting), Vertical Screen—Low Surface; SKL= Standing, Keyboard—Low Surface; SAL= Standing, Angled Screen—Low surface; SHL= Standing, Horizontal Screen—Low surface; SKH= Standing, Keyboard—High Surface; SAH= Standing, Angled Screen—High surface; SHH= Standing, Horizontal Screen—High surface; SVH= Standing, Vertical Screen—High Surface.

*Joint Angles*												
	CKL	CAL	CHL	CVL	SKH	SAH	SHH	SVH	SKL	SAL	SHL	SVL
**Right Knee**	**REF**	↑	↓	↑	---	---	---	---	---	---	---	↑
**Left Knee**	**REF**	---	---	↑	---	---	---	---	---	---	---	↑
**Right Hip**	**REF**	---	---	↓	---	---	---	---	---	---	---	↑
**Left Hip**	**REF**	---	---	↓	---	---	---	---	---	---	---	↑
**Right Shoulder**	**REF**	↑	↑	↑	↓	↓	↓	↑	↑	↓	↓	↑↑
**Left Shoulder**	**REF**	---	↑	↑	---	↓	↓	↓	---	↓	↓	↑
**Right Elbow**	**REF**	---	↑	↑	↑	↑	↑	↑	↑↑	↑↑	↑↑	↑
**Left Elbow**	**REF**	↓	↑	↓	↑	↑	↑	↑	↑↑	↑↑	↑↑	↑
**Right Wrist**	**REF**	---	---	---	---	---	---	---	---	---	---	---
**Left Wrist**	**REF**	---	---	---	---	---	---	---	---	---	---	---
**Neck**	**REF**	↓	↓	↓	---	↓	↓	↓	---	↓	↓	---
*Joint Velocities*												
	CKL	CAL	CHL	CVL	SKH	SAH	SHH	SVH	SKL	SAL	SHL	SVL
**Right Knee**	**REF**	↓	↓	↓	↑↑	↑	↑	↑	↑	↑	↑	↑↑
**Left Knee**	**REF**	↑	**---**	↓	↑	↑	↑	↑	↑	↑	↑	↑↑
**Right Hip**	**REF**	↓	**---**	↓	↑↑	↑	↑	↑	↑	↑	↑	↑
**Left Hip**	**REF**	↑	**---**	**---**	↑↑	↑	↑	↑	↑	↑	↑	↑
**Right Shoulder**	**REF**	↑	↓	↑	↑	↓	↑	**---**	↑	**---**	↓	↑
**Left Shoulder**	**REF**	**---**	**---**	↑	↑	↓	↓	↑	↓	↑	↓	↑↑
**Right Elbow**	**REF**	**---**	↓	**---**	↑	↓	↓	↓	↑	**---**	↓	↑
**Left Elbow**	**REF**	↓	↓	↓	↓	↓	↓	↓	↓	↓	↓	↑
**Right Wrist**	**REF**	↓↓	↓↓	↓	**---**	↓	↓↓	↑	↓↓	↓↓	↓↓	↑↑
**Left Wrist**	**REF**	↓↓	↓↓	↓	↓↓	↓↓	↓↓	↑↑	↓↓	↓↓	↓↓	↓
**Neck**	**REF**	↓	↓	↓	↓	↓	**---**	**---**	↑	↓	↓	↓
*Body Discomfort Measures*												
	CKL	CAL	CHL	CVL	SKH	SAH	SHH	SVH	SKL	SAL	SHL	SVL
**Upp. Extremity Discomfort**	**REF**	↓	↑	---	---	↓	↑	↑↑	↑	↑	↑	↑↑
**Low. Extremity Discomfort**	**REF**	---	---	---	---	---	↑	↑	↑	↑	↑	↑↑

Shaded outcomes indicate improvement over the reference condition.

Angle and Velocities were measured by Motus on a continuous scale (Angles and Velocities: “↑” indicates greater joint angles and velocities)

Usability scores were based on a 0–7 score, with 0 being “not applicable” and 7 being the “most usable.” (Usability: “↑” indicates that these conditions were considered “more usable”)

Discomfort scores were based on a 0–10 scale, with 0 being no pain at all, and 10 being severe pain. (Discomfort: “↑” indicates that more discomfort was experienced compared to CKL condition)

Error rate refers to the percentage of time that an error would be made within that condition. (Error Rate: “↑” indicates that more errors were made in these conditions than CKL condition)

Ranking scores were based on a 1–12 ranking, with 1 being the best and 12 being the worst. (Ranking: “↑” indicates that these conditions were ranked higher, meaning they were better).
